# Papilledema and Anemia: A Rare Association

**DOI:** 10.7759/cureus.25929

**Published:** 2022-06-14

**Authors:** Shisheer Havangi Prakash, Deepak Basavaraju, Supreeth N Gowda

**Affiliations:** 1 Internal Medicine, Mysore Medical College and Research Institute, Mysore, IND; 2 Neurology, Gopala Gowda Shantaveri Memorial Hospital, Mysore, IND

**Keywords:** raised intracranial pressure, idiopathic intracranial hypertension (iih), iron deficiency anemia (ida), anemia, papilledema

## Abstract

Papilledema is the swelling of the optic disc due to the transmission of intracranial pressure through the optic nerve. It can occur as a symptom of various intracranial pathologies that elevate the intracranial pressure. Even though anemia has been described as an association with raised intracranial pressure, the exact causal relationship between the two has not been well established in the literature. We present one such unusual case wherein a 21-year-old female patient, who complained of headaches, was found to have papilledema and severe anemia. After an unyielding workup for secondary causes, the rare association between papilledema and anemia was considered and prompt hemoglobin correction was ensued. Subsequently, resolution of papilledema and improvement in the visual acuity of the patient was observed. Although the causal relationship between anemia and papilledema has not been thoroughly explained, appropriate workup and correction of anemia play an integral role in the management of patients with papilledema and the prevention of long-term ocular complications.

## Introduction

Papilledema is defined as swelling of the optic disc due to elevation in intracranial pressure (ICP) [1]. Papilledema, in essence, is always associated with high ICP, however high ICP can exist in the absence of papilledema [2]. The etiology of high ICP can include known causes such as space-occupying lesions like tumor, abscesses, or hemorrhage; increase in CSF pressure as in meningitis or hydrocephalus; increase in the venous pressure as in cerebral venous thrombosis or it can be idiopathic. Idiopathic intracranial hypertension (IIH), the most often cause of papilledema, has an estimated annual incidence of 0.9 per 100,000 population in the United States. It most frequently affects women of childbearing age with obesity being a crucial determining factor [3]. IIH, and in turn papilledema, has also been reported as a rare association with anemia [4].

Anemia is defined as a decrease in the oxygen-carrying capacity of blood due to a reduction in the number of RBCs or the hemoglobin content in the blood. As per the World Health Organization (WHO) criteria of 2010, hemoglobin levels less than 12 g/dL in premenopausal females and 13 g/dL in postmenopausal females and males of all ages are needed for the diagnosis of anemia [5]. The relationship between anemia and IIH has not been well elaborated in literature; however, the one proposed mechanism includes a hyperviscous state, caused by anemia, which leads to increased venous pressure that in turn leads to increased ICP [6]. Here, we present a case showing this rare association between papilledema (high ICP) and anemia where a significant symptomatic resolution was observed with correction of anemia.

## Case presentation

A 21-year-old female comes to the hospital with complaints of bilateral temporal headache (right>left) of sudden onset after waking up three days prior. The headache was described as throbbing in nature, present continuously, and associated with reduced visual acuity bilaterally. It was not associated with photophobia, tinnitus, dizziness, nausea, or vomiting. She also complained of gradual onset generalized fatigability over the past three months. She had no history of trauma, medication intake, or prior pregnancy. The patient's menstrual history included regular 27-30 days cycles and 3-5 days of moderate flow.

On examination, the patient was conscious and well-oriented to time, place, and person. Her BMI was 19.5 kg/m^2^. Pallor was seen in conjunctiva, mucous membrane, and skin. She was a non-smoker, non-alcoholic with a predominantly Lacto-vegetarian diet. Her vitals on admission were as follows: blood pressure of 100/60 mm of Hg, pulse rate of 112/min, and temperature of 98.4℉ (36.8℃). On examination, visual acuity was 6/12 bilaterally with good color perception on both sides. On direct fundoscopy, optic discs were congested, hyperemic with blurring of all borders without any obscuration of blood vessels, suggesting a grade 2 papilledema. Other ophthalmological parameters were within normal limits. Further, no significant findings were present on detailed neurological, cardiac, and other systemic examinations.

In testing for possible secondary causes for raised ICP, a magnetic resonance imaging (MRI) of the brain with contrast and a magnetic resonance venogram (MRV) were ordered and no significant findings were reported. Opening pressure on lumbar puncture was found to be elevated at 270 mm of Hg, with normal cerebrospinal fluid content (CSF). The initial blood workup was positive for severe anemia hence further studies were ordered, the results of which are summarized in Tables 1, 2.

**Table 1:** Laboratory values

Laboratory investigations	Normal value	On admission
Hemoglobin (g/dL)	11.6 to 15	5.7
HCT	36.0%- 48.0%	20.6%
MCV (/fL)	80.0-99.0	52.5
MCH (/pg)	26.0-32.0	15.5
MCHC (/dL)	32.0-36.0	29.6
Serum Iron (μg/dL)	37-135	12.0
Total Iron Binding Capacity (μg/dL)	250-450	496.7
Percentage Transferrin Saturation (%)	20-55	2.4
Ferritin (ng/mL)	10.0-291.0	0.1
Vitamin B12 (pg/mL)	211-911	186.0

**Table 2:** Peripheral Blood Smear

Blood Group and Rh Typing	“O” Positive
RBCs	Decreased in number Marked anisocytosis present Microcytic, hypochromic RBCs Few elliptocytes present
WBCs	Normal in number and distribution
Platelets	Adequate
Impression	Marked microcytic, hypochromic anemia

Thyroid function test, liver function test and serum homocysteine level were unremarkable. Ultrasound of abdomen and pelvis was normal and serum beta-hCG levels were not elevated. Transthoracic echocardiogram did not show any changes associated with hyperdynamic circulation and was with normal limits. Stool routine and occult blood were within normal limits.

Based on the lab finding and the peripheral blood smear report, a diagnosis of iron deficiency anemia was made and the possible association with papilledema was considered. The patient was transfused with two pints (300 mL/pint) of cross matched and typed packed red blood cell over the course of 72 hours after admission. She was also supplemented with parenteral iron sucrose, Vitamin B12 and folic acid. Post transfusion the patient's hemoglobin levels was 9.0g/dL with a hematocrit of 26.2%. On day 5 of admission, ophthalmological evaluation was done which showed an improved visual acuity of 6/9 bilaterally and direct fundoscopy revealed no evidence of prior papilledema. The patient was discharged with oral iron, Vitamin B12 and folic acid supplementation along with nutritional rehabilitation and follow up for psychotherapy and counseling.

## Discussion

Papilledema is defined as edema of the optic nerve head due to elevated ICP. It is thought to be due to obstruction of axoplasmic transport in cells of the optic nerve due to retrograde transmission of raised ICP through the subarachnoid space. The most common cause of papilledema, in patients under 50 years of age, is due to idiopathic raise in the ICP known as IIH [7]. Friedman and Jacobson proposed a criterion that specified that all of the following criteria must be met for the diagnosis of IIH: 1) symptoms and signs of raised ICP or papilledema; 2) increased opening pressure on lumbar puncture; 3) normal CSF analysis; 4) no imaging evidence of ventriculomegaly or any structural cause for raise ICP; and 5) no other identifiable cause for intracranial hypertension [8]. The differential diagnosis for IIH includes several conditions that mimic IIH and cause raised ICP such as hydrocephalous, brain tumors, and hemorrhage. Appropriate workup must be done in order to exclude such secondary causes before the diagnosis of IIH.

Furthermore, it has been described in the literature that IIH predominately affects women of childbearing age who are more prone to be anemic due to iron-deficiency anemia or menstruation-related blood loss [3]. Although the causal association between anemia and papilledema (raised ICP) has not been well described, several anecdotal reports exist that document this relationship [4,9]. Several mechanisms have been documented suggesting the possible connection between anemia and raised ICP. One mechanism describes that the increased erythropoietin (EPO) levels in anemia can induce a positive stimulation of thrombopoiesis [10]. Based on the study of mouse models by Hacein-Bay-Abina et al., they concluded that the EPO receptor pathway acts on late-stage megakaryopoiesis and is responsible for large-sized platelet production, while the thrombopoietin pathway promotes small-sized platelet production [11]. This state of thrombocytosis can be associated with a hyperviscous and hypercoagulable state [12]. Another mechanism attributes iron deficiency to a hypercoagulable state due to a reduction in red blood cell deformability and an increase in the viscosity of blood [13].

Although IIH most commonly affects anemic menstruating women, the causation between anemia and raised ICP could not be explained by a retrospective, matched case-control study conducted by Lin et al. In their study, no statistically significant association was found between the complete blood count values in patients diagnosed with IIH compared with the controls [14]. However, there is substantial evidence in literature where symptomatic resolution of papilledema was seen with the prompt correction of anemias [4]. In one such study, IIH patients with anemia showed significant improvements in papilledema, CSF opening pressure and visual acuity after 20 days of anemia correction compared to the non-anemic group [15]. Mollan et al. reported symptomatic resolution of papilledema among seven out the eight cases upon anemia correction [16].

## Conclusions

In conclusion, clinicians should be aware of the rare relationship between anemia and papilledema. Though the casual relationship has not been proven, sufficient evidence exists warranting the need for routine workup for anemia in patients with IIH, this includes a complete blood count, serum iron and B12 studies and reticulocyte count. Further, prompt correction of anemia must ensue for symptomatic resolution and prevention of long-term ocular consequences. This report adds to the anecdotal evidence describing clinical characteristics and management of patient with papilledema and anemia.
